# Omics Approaches for the Study of Adaptive Immunity to *Staphylococcus aureus* and the Selection of Vaccine Candidates

**DOI:** 10.3390/proteomes4010011

**Published:** 2016-03-07

**Authors:** Silva Holtfreter, Julia Kolata, Sebastian Stentzel, Stephanie Bauerfeind, Frank Schmidt, Nandakumar Sundaramoorthy, Barbara M. Bröker

**Affiliations:** 1 Department of Immunology, University Medicine Greifswald, Greifswald 17475, Germany; sebastian.stentzel@uni-greifswald.de (S.S.); stephaniebauerfeind@web.de (S.B.); broeker@uni-greifswald.de (B.M.B.); 2 Department of Medical Microbiology, University Medical Center Utrecht, Utrecht 3584, The Netherlands; J.B.Kolata@umcutrecht.nl; 3 ZIK-FunGene Junior Research Group Applied Proteomics, Department of Functional Genomics, Interfaculty Institute for Genetics and Functional Genomics, EMA-University of Greifswald, Greifswald 17475, Germany; frank.schmidt@uni-Greifswald.de (F.S.); sundaramon@uni-greifswald.de (N.S.)

**Keywords:** *Staphylococcus aureus*, vaccine, immune response, adaptive immunity, genomics, proteomics, transcriptomics, immunoproteomics

## Abstract

*Staphylococcus aureus* is a dangerous pathogen both in hospitals and in the community. Due to the crisis of antibiotic resistance, there is an urgent need for new strategies to combat *S. aureus* infections, such as vaccination. Increasing our knowledge about the mechanisms of protection will be key for the successful prevention or treatment of *S. aureus* invasion. Omics technologies generate a comprehensive picture of the physiological and pathophysiological processes within cells, tissues, organs, organisms and even populations. This review provides an overview of the contribution of genomics, transcriptomics, proteomics, metabolomics and immunoproteomics to the current understanding of *S. aureus*‑host interaction, with a focus on the adaptive immune response to the microorganism. While antibody responses during colonization and infection have been analyzed in detail using immunoproteomics, the full potential of omics technologies has not been tapped yet in terms of T-cells. Omics technologies promise to speed up vaccine development by enabling reverse vaccinology approaches. In consequence, omics technologies are powerful tools for deepening our understanding of the “superbug” *S. aureus* and for improving its control.

## 1. Introduction

*Staphylococcus (S.) aureus* is a Janus-faced microorganism: On the one hand, about 20% of human adult population are persistently colonized with this bacterium, usually without clinical symptoms [1]. On the other hand, *S. aureus* is notorious for causing a broad range of infections and for rapidly evolving resistances [1,2]. These bacteria are the second most common cause of hospital-acquired infections in general and the leading cause of skin and soft tissue infections, nosocomial pneumonia, wound infections, and Gram-positive sepsis in particular [3,4,5]. 

The high incidence of *S. aureus* infections is due to the expression of a broad variety of bacterial virulence and immune evasion factors and to the rapidly evolving resistance to antibiotics [2,4,6,7,8]. Methicillin-resistant *S. aureus* (MRSA) are spreading in hospitals as well as in the community [4,9], and *S. aureus* strains resistant to last reserve antibiotics are reported worldwide. Worryingly, no new classes of antibiotics have been introduced to the market by the pharmaceutical industry over the last three decades. Hence, we may be facing a future where *S. aureus* can not be treated efficiently anymore [10]. These alarming perspectives are calling for additional preventive and therapeutic strategies, such as vaccination and novel anti-microbial therapies. To date, however, all *S. aureus* vaccine trials have failed [11,12,13].

Omics technologies provide panoramic views of the molecular determinants of life and their interactions enabling an unbiased approach to physiological and pathological processes. The methods (with the exception of metabolomics) are grounded in genomics, which was sparked by the sequencing of the complete genome of the bacterium *Haemophilus influenzae* in the year 1995 [14]. The successful deciphering of the human genome in the year 2001 marks another milestone [15,16]. Omics studies generate detailed and comprehensive insights on the information content of DNA (genomics), its temporal transcription into RNA (transcriptomics), and its translation into proteins (proteomics) and metabolites (metabolomics). Furthermore, immunoproteomics provides an overview of immunogenic peptides or proteins. The resulting broad perspective can complement targeted strategies that aim at elucidating the functions of single factors in cause and effect chains. 

Previous attempts to translate promising pre-clinical results into a successful vaccine for patients have given disappointing results. Omics technologies are a powerful tool to help overcome the hurdles by increasing our knowledge about the mechanisms of protection. This review starts with a brief outline of current challenges in studying adaptive immunity to *S. aureus.* Following this, it provides an overview of the contributions genomics, transcriptomics, proteomics, metabolomics and immunoproteomics to our current understanding of:

the *in vivo* behavior of *S. aureus*;
the antibody and T-cell response against *S. aureus*; and
vaccination development.

## 2. Challenges in Deciphering the Adaptive Immune Response to *S. aureus*

In order to be able to prevent or treat *S. aureus* infections in the future, we need to learn more about the *in vivo* behavior of these bacteria and the immunological mechanisms of protection. However, such studies are impeded by various factors, such as:

the diversity and complexity of *S. aureus* host interactions;
the impressive genetic variability of the species *S. aureus*;
a deficit in infection-relevant *in vitro* and *in vivo* models; and
the high variability of the anti-staphylococcal immune responses.

Firstly, *S. aureus* host interactions are multifaceted. On the one hand, *S. aureus* is a frequent colonizer of the human skin and mucosa: Around 20% of adults are persistent carriers of this microorganism; the others are intermittently colonized [17]. However, intensive exposure to *S. aureus* in carriers is a risk factor for infection upon hospitalization [18,19], which in most cases is caused by the colonizing *S. aureus* strain [20,21]. On the other hand, *S. aureus* is a prominent pathogen [1,2], causing skin and soft tissue infections, wound infections, osteomyelitis, pneumonia, and sepsis [3,4,5]. For decades, *S. aureus* has been considered a classical extracellular pathogen. However, it has become evident that *S. aureus* can survive in different types of non-professional phagocytes, such as epithelial and endothelial cells [22,23]. This ability to invade host cells, to escape from the lysosomal degradation machinery and to persist within the intracellular location most likely contributes to long-term persistence and recurrent infections [22]. Both the bacterial behavior and the mechanisms of protection probably depend critically on the type of infection.

Secondly, *S. aureus* research is challenged by the genetic variability of the species. Two strains can differ in up to 25% of their gene content [24,25]. Hence, data obtained with a single strain cannot be easily generalized. This species variability fuels a continuous discussion as to whether conserved or variable (but disease-specific) antigens should serve as vaccine targets. There is consensus that a multivalent vaccine that reflects the genetic diversity of the *S. aureus* species will be superior to a monovalent vaccine [26,27,28]. 

Thirdly, the characterization of infection-related properties of *S. aureus* is currently hindered by a lack of data generated from *in vitro* and *in vivo* models that closely mimic infection in humans. Bacterial behavior has been studied extensively in very simplified systems, e.g., by adding stressors to broth cultures. There is an increasing awareness that *in vivo* virulence regulation can differ substantially from that seen *in vitro* [29,30,31,32]. Therefore, the focus is now shifting to more complex model systems, such as cell cultures, animal models, and human samples. This shift is promoted by advances in omics techniques. Additionally, recent studies have highlighted the host-specificity of numerous virulence factors and evasion molecules, which needs to be considered when choosing the appropriate animal model [33,34,35,36,37].

Finally, understanding adaptive immunity to *S. aureus* is challenged by the variability of the human host and the induced adaptive immune responses. Adaptive immunity in each individual is shaped by the type of *S. aureus* strain and the micro-environmental context in which the host encounters the bacteria (reviewed in [38]). Therefore, it is of no surprise that the antibody patterns within the human population are highly variable, regarding both specificities and titers [39,40,41]. Compared to antibodies, relatively little is known about specific T-cell responses against *S. aureus* in humans [38]. 

## 3. Omics Technologies in *S. aureus* Research 

The remarkable success of *S. aureus* as a colonizer and pathogen depends on its ability to adapt to different environments, e.g., abiotic surfaces such as prosthetic implants and catheters [42,43], the mucosa of the nose, the gastrointestinal and reproductive tract, as well as—upon invasion—virtually every human tissue [1]. Survival in such a wide variety of environmental niches requires adaptation responses, which can affect all regulatory levels from gene to transcript, protein and metabolite. Omics technologies provide powerful tools to study *S. aureus* pathophysiology and host interaction in its entirety and complexity in different experimental set-ups such as cell cultures, animal models, and human samples (Table 1). 

### 3.1. Genomics

Understanding of adaptive immunity to *S. aureus* requires knowledge about the spatial and temporal prevalence of virulence genes in the *S. aureus* population as well as about virulence gene sequence variability. 

This area of research has benefitted greatly from whole genome sequencing and DNA microarrays, which have revealed an impressive genetic diversity of the species *S. aureus*. Around 25% of the 2600 genes of *S. aureus* are variable, encompassing the so-called core variable and the variable subgenomes [25]. The core-variable genome accounts for 10% of the genome and comprises, for example, lineage-specific variants of surface adhesions and regulators. Another 15% of the staphylococcal genes belong to the variable genome. These genes are located on mobile genetic elements, such as phages, plasmids and pathogenicity islands [24]. The encoded variable subproteomes are enriched in virulence factors and are therefore highly relevant for host pathogen interaction [24,25]. Whole genome sequencing has been very successfully applied to study *S. aureus* evolution, even in a single individual during colonization or infection, as well as bacterial transmission during outbreaks [44,45,46,47,48,49,50,51]. By comparing virulence gene contents of *S. aureus* isolates from different clinical cohorts and the general population, comparative genomics can indicate targets for vaccination but also provide insights into the molecular basis of pathogenicity [52].

Based on genome information, DNA microarrays have been developed. They have provided valuable insights into the genetic variability of *S. aureus* in the general population, in hospital settings and defined patients groups [53,54,55]. Due to their rapid and simple handling and straightforward data analyses, custom or commercial DNA microarrays are still widely used to obtain an overview of the repertoire of antibiotic resistance and virulence genes [56,57,58,59,60,61,62,63]. However, in comparison to next-generation sequencing, DNA microarrays are of lower resolution, and they do not allow the identification of unknown virulence factors. 

From a host perspective, genome-wide association studies uncover host gene polymorphisms associated with bacterial colonization or certain clinical pictures. The state-of-the-art host genetic susceptibility to *S. aureus* carriage and infections has recently been reviewed by Shukla and colleagues [64]. Most investigations into host genetic determinants of *S. aureus* nasal carriage used a candidate gene approach [65,66,67,68]. A few studies tried to identify host polymorphisms associated with *S. aureus* disease on a genome-wide level but failed [69,70,71]. Future investigations using larger sample numbers and narrowed phenotypes as well as building on advances in both genotyping and analytical methodologies will offer the chance of identifying new genetic variants important for *S. aureus* colonization and infections. 

Focusing on the immune defense, several elegant methods are available to decipher the T-cell receptor and B-cell receptor repertoires [72,73,74,75]. They are based on next-generation sequencing and have already been used for investigations of the T-cell receptor repertoire in cancer, autoimmune diseases and viral infections. In terms of TCR sequencing upon bacterial stimulation, Li *et al.* have profiled the TCR repertoire in patients with pleural tuberculosis [76]. Diluvio *et al.* utilized this method to confirm that, in patients suffering from psoriasis vulgaris, certain TCR beta-chain variable region (TCRBV) genes are clonally expanded in the skin lesions. Interestingly, if these patients additionally developed streptococcal angina, tonsillar T-cells with identical TCRBV genes as in the psoriatic skin lesion were clonally expanded [77]. For addressing the spectrum of *S. aureus-*specific T-cells, T-cell receptor sequencing has not been employed so far.

Concerning rational vaccine design, pan-genomics and comparative genomics enable a novel approach to vaccine development termed reverse vaccinology, an unbiased discovery process for candidate vaccine antigens (see Section 4). 

### 3.2. Transcriptomics 

High-throughput transcriptomics can reveal changes in gene expression profiles of both the pathogen and the host cells under infection-related conditions as well as during infection. This information is relevant for studying adaptive immune responses to *S. aureus* and also for vaccine development, because only those antigens that are actually expressed *in vivo* are vaccine candidates.

Transcriptomes of bacteria and host cells can be profiled using microarrays as well as next-generation RNA sequencing technologies (RNAseq) (Table 1). Current microarrays contain millions of probes on a single chip, enabling the simultaneous measurement of gene expression of a multitude of genes. Microarray analyses for studying *S. aureus* host interaction *in vitro* require sufficient starting material, *i.e.*, around 5 µg RNA, which corresponds to 5 × 10^5^ host cells and 1 × 10^8^ bacterial cells. RNAseq employs DNA deep sequencing technology to sequence all RNA transcripts within a sample. The technique is rapidly replacing traditional expression microarrays as the method of choice for determining global gene expression profiles in bacteria and host [78,79,80,81]. It requires less material than RNA microarray approaches, and provides the possibility to determine absolute transcript levels of sequenced but also of non-sequenced organisms. Moreover, this method is able to identify novel transcripts and RNA isoforms. For validating data from microarray and RNAseq experiments, qRT-PCR is the method of choice. 

In the past, great efforts have been made toward the mapping of transcriptomes of *S. aureus* laboratory strains and clinical isolates under environmental stressors in cell culture, such as aerobic *versus* anaerobic growth, antibiotics, oxygen radicals, nitric oxide or iron limitation [82,83,84,85,86,87]. However, recent data has shown that the *S. aureus* transcriptional profile during growth in broth culture may correlate poorly with gene expression in mammalian colonization and infection models [29,30,31,32]. Therefore, the focus is now shifting to more complex model systems that mimic the *S. aureus* host interaction more closely. For example, transcriptome analyses were performed on *S. aureus* cells grown in body fluids, *i.e.*, serum, blood, pulmonary surfactant, artificial nasal secretions or sputum medium [88,89,90,91,92]. Moreover, transcriptomics provides insight into transcriptional adaptation to different staphylococcal lifestyles, such as intracellular survival in professional or nonprofessional phagocytes, small colony variants (SCVs) and biofilm formation [93,94,95]. 

So far, most studies have analyzed transcription profiles of either bacterial cells [95,96] or host cells [97,98,99,100,101,102,103,104], while studies combining both biological systems are still rare. In a pilot study, Depke studied host gene expression in a kidney abscess model using transcriptomics and simultaneously monitored the expression of selected *S. aureus* genes by RT-qPCR [105]. Regarding the adaptive immune response to *S. aureus*, especially that of T lymphocytes, transcriptomics has been limited to the investigation of certain target genes by real-time-PCR [106,107,108,109,110]. In our group, T-cells of healthy individuals were isolated from PBMCs, stimulated with recombinant *S. aureus* antigens, and the transcription profiles of the resulting T-cell lines were analyzed using array technology. In line with the data from mouse experiments, these cells predominantly but not solely responded in a Th1/Th17 manner (Steinke, personal communication). 

The grail, however, is the study of gene regulation and gene expression in real life, namely, infected or colonized animals or patient material. Characterizing host responses during resolution of *S. aureus* infection will allow the definition of immune correlates of protection. The amount of RNA required and the vast excess of host RNA over bacterial RNA in most infected tissues are limiting the application transcription profiling to infections with low bacterial densities or asymptomatic colonization. Gene expression analysis during persistent colonization has been studied using quantitative RT-PCR as well as recently via RNASeq [29,81,111]. The data of the direct *ex vivo* RNASeq approach corroborate results obtained with an artificial nasal medium and show that the nasal micro-environment imposes iron and nutrient limitation stress upon the bacteria [81,112]. Initial studies addressing the *S. aureus* transcriptome during infection [32,113,114,115] suggest that each infection type, e.g., pneumonia, endocarditis or abscesses, has its specific signature, due to the presence of organ-specific environmental triggers [86,116]. 

### 3.3. Proteomics 

Proteomics approaches include 2D-gel-based proteomics, shotgun-proteomics, as well as protein microarrays (Table 1). 2D-gel-based proteomics was developed in the 1990s and combines protein separation (2D-PAGE), enzymatic protein digestion, detection of the resulting peptides by MALDI-MS and the bioinformatic analysis of the resulting peptide mass fingerprints (PMF). Moreover, fluorescent dyes enable sensitive in-gel detection of proteins and their quantification over a wide dynamic range [117]. Though the high resolution of protein species, *i.e.*, protein variants encoded by a single gene, is of advantage, this method is limited due to the high manual effort, the required sample size and the selective analytical window of the targeted proteins. 2D-gel-based proteomics has been instrumental in the analysis of the *S. aureus* proteome [118].

The rapid development of MS techniques with high mass accuracy in combination with novel gel-free sample preparation methods enabled gel-free proteomics, which is superior in terms of comprehensiveness of the acquired data, versatility of the accessible samples, sensitivity, resolution and the required protein amount (ng *vs.* µg quantities) [119,120]. Moreover, quantitative proteomics workflows have been implemented using isotopic labeling or even label-free analyses [121].

When applying proteomics to the analysis of pathogen and host, it is—in contrast to the analytics of nucleic acids that can cover the entire genome—usually impossible to catch the entire proteome in one single protein preparation. Cytosolic, membrane and membrane-associated, cell surface-exposed, and secreted proteins need to be analyzed separately [120,122,123]. Using sophisticated methods for the sub-fractionation of the staphylococcal subproteomes, Becher *et al.* were able to provide a very comprehensive protein inventory of living bacteria, including quantitative data for almost 1700 *S. aureus* proteins, corresponding to 80% coverage of all expressed proteins [118]. Highlighting the technical advances in MS, nowadays even unfractionated protein samples may reach an identification rate of roughly 90% by using high sensitivity mass spectrometry combined with sophisticated peptide enrichment techniques, as shown by Depke *et al.* [124].

Over the last 15 years, one focus of proteomics application to *S. aureus* research has been on understanding the functional adaptation to stress and starvation as they are encountered by the bacteria during infection and/or antibiotic treatment [120,125,126,127]. Engelmann and colleagues defined proteome signatures of different stressors, including fermentation, nitrate respiration, diamide stress, H_2_O_2_, and nitric oxide [125,128,129,130]. These signature libraries form a useful toolbox for deciphering the physiological state of bacteria grown under infection-related conditions [120,131,132]. Moreover, they can aid in finding key enzymes and therefore potential key targets for novel antimicrobial therapies [120]. The proteome signature data are publicly available in the database Aureolib [132].

Cell surface-exposed secreted bacterial proteins are subproteomes that are centrally involved in *S. aureus* host interaction. They are enriched for virulence factors, immune evasins and adhesins [123], which are predominantly targeted by the humoral immune response and thus represent candidates for antibody-based vaccines [133]. By dissecting the exoproteomes of 25 clinical *S. aureus* isolates, Ziebandt *et al.* discovered that their composition was extremely variable; only eight proteins were shared by all isolates [134]. This variability was only partially explained by genome plasticity but mainly resulted from a high degree of expression heterogeneity. These data highlight the importance of combining different omics approaches to obtain a complete picture of the bacterial behavior [134]. An important issue to be addressed in future studies will be the identification disease-specific (exo)proteome signatures as they have been reported in a recent pilot study [135]. 

*In vivo* proteomics studies analyzing the functional state of *S. aureus* during colonization or infection are still rare, because they are hampered by the minute amounts of sample that can be obtained *in vivo*, as well as by the interference by abundant host materials like proteins and/or nucleic acids [120,136]. In order to enrich bacteria from *in vitro* infection experiments, approaches to separate bacteria from host cells include centrifugation, immune-magnetic separation and fluorescence-activated cell sorting (FACS) [137,138,139,140]. The isolation of intracellular compartments such as phagosomes, which contain the bacteria and their secretion products, promises the identification of bacterial proteins that are released by the bacteria during invasion and persistence inside host cells [140,141,142,143]. Using a FACS-based enrichment protocol for quantitative proteome profiling of internalized *S. aureus* in human airway epithelial cells [119], almost 1500 *S. aureus* proteins were identified with highly sensitive MS equipment from only 2 × 10^6^ bacterial cells [139]. Furthermore, we recently developed a workflow for the simultaneous assessment of both the bacterial and the host cell proteomes (using SILAC quantification), showing that intra-cellular persisting bacteria were growing slowly, induced the stringent response, and had to cope with microaerobic conditions as well as with cell wall stress [144]. A pilot proteomics study on murine *S. aureus* pneumonia demonstrates that proteomics are feasible for *ex vivo* samples, such as bronchoalveolar lavages [145]. 

Focusing on the host immune defense, proteomics, especially when combined with next-generation sequencing [146], opens new avenues for the elucidation of the antibody- and T-cell repertoires [147,148]. This technology has not yet been applied to comprehensively map the T response to *S. aureus*. On a smaller scale, T-cell function is usually addressed by determining the generation of cyto- and chemokines as well as the expression of cell surface markers using multiplex assays. The human cytokinome, comprising all known cytokines and chemokines, has more than 240 members [149]. It is currently not possible to record the whole cytokinome at once due to the large dynamic concentration range of different cytokines. However, advanced bead-based methods are available to simultaneously measure approximately 50 cytokines/chemokines from a single sample [150]. When addressing the antigen-specific cytokine response of T-cells to *S. aureus*, such bead-based multiplex assays are currently the method of choice [151,152]. 

Cell surface marker expression can be addressed by advanced flow cytometric approaches as well as mass cytometry (e.g., CyTOF technology), enabling the simultaneous assessment of up to 40 parameters. These multiplex approaches allow the analysis of T-cell differentiation upon antigen-specific activation. State-of-the-art mass spectrometry applied to extracts enriched in cell surface proteins could in the future help to resolve the modulation of the T-cell surfacome in *S. aureus* infection in its full complexity. 

### 3.4. Immunoproteomics 

Immunoproteomics is a sub-discipline of immunomics, which aims at studying the function and regulation of the immune system in its entirety using omics approaches [147,148,153,154,155]. Immunoproteomics builds on proteomics for the comprehensive analysis of the adaptive immune response. Using gel-, array-, and mass spectrometry-based techniques, immunoproteomics has the goal of identifying and measuring antigenic peptides or proteins as well as the adaptive immune response directed against them [156,157].

**Figure 1:**
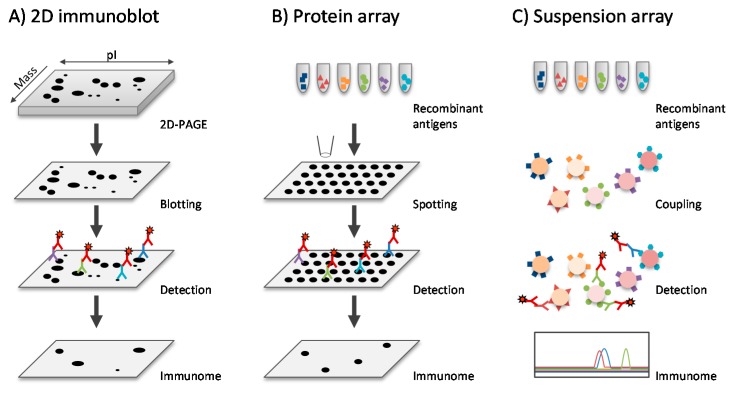
Workflow of immunoproteomics approaches**.** Schematic representation of three commonly used immunoproteomics-based approaches for the identification and quantification of anti-staphylococcal antibodies. (**A**) 2D-immunoblots. *S. aureus* proteins are separated based on their pI, followed by gel-based resolution according to their molecular weight. Afterwards, proteins are transferred to a membrane by Western blotting and immobilized. Anti-*S. aureus* antibodies from patient sera specifically bind to their respective *S. aureus* antigen and are visualized by labeled secondary antibodies. Since the bacterial antigens are denatured during resolution on 2D gels, predominantly non-conformational epitopes are detected with this approach; (**B**) Protein Array. A panel of recombinant or purified *S. aureus* antigens is spotted on a solid surface in an ordered manner. Afterwards, anti-*S. aureus* antibodies in patient sera are detected using labeled secondary antibodies. Proteins can be applied in their native form, allowing the detection of conformational epitopes; (**C**) Suspension array. Up to 500 discrete assays are performed simultaneously on the surface of distinct color-coded beads known as microspheres. Using multiple lasers or LEDs and high-speed digital-signal processors, an analyzer reads multiplex assay results by reporting the reactions occurring on each individual microsphere. For *S. aureus* immunoproteomics, panels of recombinant or purified *S. aureus* antigens have been coupled to distinct microspheres, and anti-*S. aureus* antibodies can be quantified over a large linear range after incubation with patient serum and labeled secondary antibodies. Additionally, in this case, antigens with conformational epitopes can be detected, if proteins are coupled in their native conformation. Images were adapted from Tjalsma *et al.* [159].

For unbiased anti-staphylococcal antibody profiling, 2D-immunoblotting (2D-IB) in combination with MS has been employed and the immunogenic antigens of *S. aureus* have been mapped (Figure 1). While being labor-intensive, it enables a personalized approach by simultaneously providing information about the virulence factor repertoire of a clinical *S. aureus* isolate (proteome) and the specific antibody response of the affected patient or carrier (immunoproteome) [39,40]. In view of the pronounced heterogeneity of the species *S. aureus* a personalized strategy reduces the experimental noise, and it has revealed rules governing the antibody response to *S. aureus* [38,157,158]. 

Immunocapture MS has a similar scope [159]. This technique is based on immobilization of patient antibodies, which are directly used to isolate antigenic proteins from a complex mixture of proteins. The captured antigens are subsequently profiled by MS. This method allows the detection of conformational epitopes, because, in contrast to 2D-IB, non-denatured protein mixtures can be exposed to the immobilized antibodies. 

Finally, *E. coli* surface display libraries have been established for identifying *S. aureus* antigens that are recognized by antibodies [160]. Here, either uniformly small (linear epitopes) or uniformly medium-sized (potential conformational epitopes) peptides encoded by the bacterial genome are displayed on the surface of *E. coli* via fusion to outer membrane proteins. The resulting *E. coli* libraries can be probed with patient sera.

Building on prior knowledge about the antigenic composition of *S. aureus*, immunogenic *S. aureus* proteins can be recombinantly expressed for (1) validation of their role as prominent antibody targets and (2) integration into multiplex assays permitting high throughput quantification of specific antibodies (Table 1) [41,133,161,162]. Most multiplex assays used in *S. aureus* research are based on suspension array technology (e.g., Luminex^®^) that allows simultaneous quantification of antibody binding to up to 500 bacterial proteins over a large dynamic range (10^5). Alternatively, *S. aureus* proteins or peptides can be spotted onto protein arrays (Figure 1) [159,163]. 

#### 3.4.1. Antibody Profiles in Healthy Individuals 

Healthy individuals, be they *S. aureus* carriers or noncarriers, harbor antibodies against a broad spectrum of *S. aureus* antigens (as reviewed in [39]). As expected, antigens accessible at the cell surface or released by the bacteria are immunodominant over intracellular proteins. The antibody spectrum within the human population is highly variable, regarding both specificities and titers [39,40,41], which reflects the history of encounters with *S. aureus*. For example, multiplexed bead-based assays demonstrated that healthy individuals, be they carriers or non-carriers, mount a highly variable antibody response against *S. aureus* surface and secreted proteins with titers differing by a factor of 1:10 to 1:1000, depending on the tested staphylococcal antigen [41,164] (and Nandakumar Sundaramoorthy, personal communication).

How are these serum antibodies induced? Studies suggest that mere epithelial colonization is not sufficient to trigger a serum IgG response in *S. aureus* carriers. For example, nasal colonization in infants frequently precedes sero-conversion, both the generation of IgM and IgG, for many months [165]. Moreover, experimental nasal colonization in humans does not elicit a robust serum IgG response [40]. This suggests that the specific immune memory of *S. aureus* observed in most adults is probably elicited by minor invasive episodes. 

The high level of antibodies in noncarriers shows that these persons are also frequently exposed to *S. aureus*. In fact, when measuring the antibody response to conserved *S. aureus* antigens, the differences between carriers and non-carriers are small in comparison to the very large variations within the two groups [41,165,166]. However, while carriers will experience repeated episodes of infection with their colonizing strain, resulting in a strong strain-specific immune response [167], non-carriers might contact a wider range of different *S. aureus* isolates. In line with this, healthy carriers have a robust antibody response to *S. aureus*, which is even slightly stronger than that in non-carriers [39,41,164,165]. It is obvious that antibodies do not eliminate *S. aureus* from the body surfaces; there is no sterile immunity to *S. aureus*. Nor do these antibodies reliably protect from infection [18,19] or from colonization with a different *S. aureus* strain [168].

#### 3.4.2. Antibody Profiles during Infection 

*S. aureus*-specific antibody patterns in the general population are highly variable. Thus, persons infected by *S. aureus* will likely be at very different immunological “starting positions,” which might influence the outcome. In line with this, recent data suggest that intensive exposure protects from severe *S. aureus* disease and death. While carriers acquire an *S. aureus* bacteremia more frequently than non-carriers, mostly from their endogenous strain, they have a significantly better chance of survival [18,19]. To explain this, we have proposed that long-term exposure to *S. aureus* in carriers primes the adaptive immune system, likely via repeated subclinical skin infections [39,167]. Indeed, using the highly variable superantigens as strain-specific indicator antigens, our group demonstrated that the antibody response in healthy carriers is highly specific for their colonizing strain [167]. In line with this, patients with failure of skin barriers, such as epidermolysis bullosa, are colonized with *S. aureus* at very high density and have unusually high amounts of anti-*S. aureus* antibodies in their bodily fluids. In spite of their chronic skin wounds, these patients rarely develop life-threatening systemic infection [169,170]. This supports the notion of adaptive immune protection.

Since antibody responses to *S. aureus* are partially strain-specific and *S. aureus* strains differ greatly in their repertoire of secreted antigens, Kolata *et al.* used a personalized approach to analyze the antibody response during *S. aureus* bacteremia. Using 2D-gel-based immunoproteomics the prospective study revealed that *S. aureus* carriers had established a specific IgG response to their colonizing *S. aureus* isolate already before infection onset [158]. In the case of bacteremia with their own endogenous strain, this pre-existing memory response was boosted, and IgG titers increased [158]. In contrast, non-carriers infected with an exogenous *S. aureus* strain, started from lower basal antibody levels, presumably because the immune systems had not previously been exposed to the invasive *S. aureus* isolate. Over the course of bacteremia, their antibody patterns were drastically altered with the acquisition of many new specificities and increases of titers [158]. An immunoproteome signature of 11 conserved *S. aureus* proteins was defined, and the proteins were recognized by antibodies in at least half of the bacteremic patients [158]. Using a similar approach, immunoproteome signatures of *S. aureus* colonization, skin and soft tissue infection, and bacteremia have been reported by Liew and colleagues [135]. Further studies of well-defined patient cohorts are urgently required to identify disease-specific immunoproteome signatures with diagnostic potential.

Prospective clinical studies may also reveal immune parameters predictive of disease outcome, as shown in a pilot study. By combining two immunoproteomic assays, *i.e.*, automated 1D-immunoblots and suspension arrays, *S. aureus* bacteremia patients could be stratified according to their risk of developing sepsis, and IgG specificities that can serve as a marker for protection from sepsis were identified [133]. Hence, the immunological “starting position,” seems to be important for disease outcome, which encourages efforts in vaccine design. 

#### 3.4.3. T-cell Responses to *S. aureus*

Compared to antibodies, less is known about the specificity of *S. aureus*-reactive T-cells. The cellular arm of the adaptive immune system merits attention, because effector T-cells decisively influence the innate and the adaptive immune response: Depending on their differentiation into helper T-cell subpopulations such as Th1, Th2, Th17 or Treg cells, T-cells shape the Ig (sub)class composition, support the formation of memory B-cells, and/or enforce the recruitment of neutrophils [171,172]. The broad repertoire of *S. aureus*-specific B-cells points to a large pool of *S. aureus*-specific T-cells, because most B-cell responses rely on T-cell help [38]. Moreover, certain subsets of T-cells are required for an efficient and fast clearance of invading *S. aureus* [173,174]. In particular, Th17 cells were found to play a protective role in different animal models of *S. aureus* infection, most prominently in cutaneous infection [175,176,177,178,179]. Additionally, the current knowledge about intracellularly persisting staphylococci suggests that *S. aureus*-specific CD8+ T-cells may play a role in the adaptive immune response to *S. aureus* as well [180]. 

The comprehensive analysis of the T-cell antigen repertoire of *S. aureus* is a challenging task for several reasons: First, antigen-specific T-cell activation has stringent requirements involving antigen processing and presentation by host cells. Second, *S. aureus* releases virulence factors that interfere with standard T-cell activation assays; superantigens activate large subpopulations of T-cells independent of their antigen specicity and override the antigen-specific responses in cell culture [181,182]. Moreover, toxins such as α-toxin or leukocidins kill T-cells [183,184]. Hence, T-cell immunoproteomics has to build on prior knowledge about putative immunogenic antigens derived either from genome information or from the analysis of the antibody response. The specific T-cell repertoire can then be probed with recombinant *S. aureus* proteins. Applying this approach to the analysis of ten healthy adults, the *S. aureus*–specific T-cell pool was estimated to comprise 3.6% of the peripheral blood T-cells with an astounding 35-fold difference between individuals (range 0.2%–5.7%) [152]. The *S. aureus* antigen-reactive memory T-cells will probably influence the course of *S. aureus* infection.

Other comprehensive methods to identify T-cell epitopes include MHC microarrays and *in-silico* analyses. MHC microarrays use peptide-MHC complexes in combination with co-stimulatory molecules as probes and T-cell populations as targets and can map MHC-restricted T-cell epitopes [185]. However, such techniques have not been applied to the *S. aureus* T-cell immunome yet.

### 3.5. Metabolomics 

Metabolome analyses comprehensively characterize the low molecular metabolites (<1 kDa), which occur in a cell in an impressive number (several thousand different molecular species) and chemical diversity. The two most important detection methods are MS and nuclear magnetic resonance (NMR) spectroscopy (Table 1) [186]. NMR spectroscopy is “non-destructive,” *i.e.*, biosamples such as urine or blood plasma can be analyzed without further sample preparation. However, it is relatively insensitive. For lower concentrations, mass spectrometry is the method of choice. It is often combined with different separation techniques such as gas chromatography, liquid chromatography and capillary electrophoresis. Substrate identification and absolute quantification is carried out by a comparison with mass spectral fingerprint libraries and reference standards. 

There are a number of reasons why the metabolome is relevant for studying adaptive immune responses to *S. aureus* and for vaccine development. Firstly, metabolic processes in *S. aureus* are linked to virulence and invasive capabilities [186]. Secondly, the elucidation of the *in vivo* metabolism of *S. aureus* can lead to the identification of new antimicrobial targets and compounds [112]. Finally, there is evidence that the metabolic state of the host influences the adaptive immune response [187]. 

Metabolomics is a young research field. First studies show the potential of these technologies [112,188,189,190,191]. For example, Krismer *et al.* used a combined metabolomics and transcriptomics approach to explore the adaptation of *S. aureus* during colonization of the human nose [112]. They observed that the methionine biosynthetic pathway is strongly upregulated and hence represents an interesting antimicrobial target.

Particularly promising, but technically very demanding, is the direct analysis of samples from infected hosts (*in vivo* metabolomics). The metabolic profiles of bodily fluids (serum, urine) are expected to reflect the molecules generated by the host immune response and non-immune cells that are directly affected by the disease, as well as by the pathogen [192,193]. Hence, metabolome studies will not only help to understand host pathogen interaction during infection, but can also aid in finding novel approaches for diagnosis and treatment, including potential vaccine targets. 

## 4. Omics Technologies in *S. aureus* Vaccine Development

**Figure 2:**
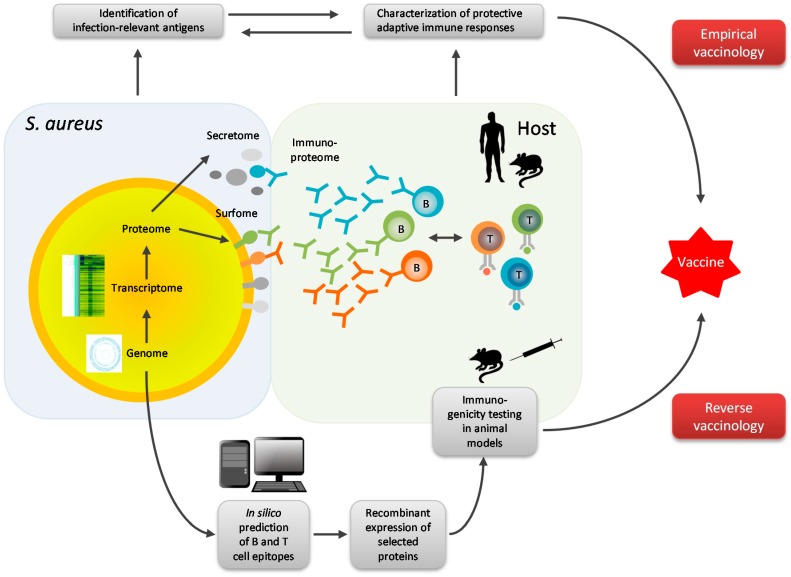
Combined approaches to successful *S. aureus* vaccine development. In empirical vaccinology, infection-relevant *S. aureus* antigens can be comprehensively mapped using transcriptomics and proteomics. In parallel, immunoproteomics provides a panoramic view of the intensity and dynamics of antibody binding to *S. aureus* proteins revealing their immunogenicity. Moreover, omics technologies can aid the characterization of the T-cell response to *S. aureus* in its full complexity. These empirical approaches will lead to the discovery of promising *S. aureus* vaccine candidates. Reverse vaccinology is a genome-based unbiased discovery process for candidate vaccine antigens. First, the whole *S. aureus* genome is mined for potential B- and T-cell epitopes using computer-based algorithms. Next, candidate antigens are produced as recombinant proteins and purified. These antigens as well as those that have been identified empirically are then used for vaccination in pre-clinical infection models and assayed for their ability to mediate protection. Promising candidate vaccines will then be subjected to clinical trials (not shown). Hence, omics technologies are versatile tools empowering both empirical and *in-silico*-based vaccine development.

The omics revolution, including novel bioinformatics tools for data analysis, has extended the options in vaccine research beyond empirical strategies, promising to speed up vaccine development. It enables an approach termed “reverse vaccinology,” a genome-based unbiased discovery process for candidate vaccine antigens (Figure 2) [194,195,196,197]. Rather than starting from live attenuated or inactivated microorganisms or drawing on prior knowledge about pathogen-host interaction, reverse vaccinology begins with an analysis of the microbial genome for open reading frames to reveal the putative proteome. This analysis can be refined in several ways to narrow down the number of candidate antigens to be tested in pre-clinical models: (1) Computational comparison will reveal the degree of protein conservation within and between microbial species; (2) tools predicting subcellular localization can be used for filtering out molecules accessible to antibodies, namely, proteins released by the microorganism or expressed on its surface; and (3) there are algorithms predicting T-cell and B-cell epitopes and hence immunogenicity [197,198,199,200,201,202]. Recombinant expression of the *in-silico* selected vaccine candidates and testing them for immunogenicity and protection in pre-clinical models are then the next steps (Figure 2) [194]. 

With the intention of further reducing the number of microbial antigens to be examined, computer aided selection tools have been developed that draw on available information about successful vaccine antigens to deduce common features, such as chemical properties of amino acid sequences [203] or functional domains [204], and apply this knowledge to the discovery of vaccine candidates in microbial genome databases. The latter approach, termed protectome analysis, is based on the notion that protective vaccines should target bacterial virulence factors that are dangerous for the host and hence share biological functions in addition to being immunogenic [204]. 

In a pilot study, Oprea *et al.* employed a simplified reverse vaccinology approach to identify *S. aureus* candidate epitopes that induce both B- and T-cell mediated immunity [205]. Instead of starting with the whole bacterial genome, they selected ten conserved surface exposed proteins for antigenicity testing and identified epitopes from fibronectin binding protein A (FnbpA), collagen adhesion (Cna), serine-rich adhesin for platelets (SraP) and elastine binding protein (EbpS) as putative targets. These, however, still need to be validated [205]. 

On the other hand, omics technologies are also empowering empirical approaches to vaccine development (Figure 2). They can be applied to the study of preclinical models but also of vaccine target populations directly. Given the fact that successful vaccination in mice could not yet be translated into an effective human vaccine, this is a big advantage. As shown above, the natural human immune response to *S. aureus* colonization and infection can now be mapped with unprecedented completeness and resolution [157]. Immunoproteomics provides a panoramic view of the intensity and dynamics of antibody binding to *S. aureus* proteins revealing their immunogenicity [40,158,206,207]. Antibody profiling in patient cohorts by multiplex assays is useful for hypothesis testing as well as for hypothesis generation [133,169,208,209,210]. It leads to the discovery of promising vaccine candidates such as the small set of *S. aureus* antigens, whose recognition by antibodies was associated with protection from sepsis in *S. aureus* bacteremia patients [133]. Finally, antibody binding is a good lead for T-cell antigen selection, since the development of high affinity antibodies requires T-cell help in most cases [152]. For targeting *S. aureus* persisting inside host cells comprehensive information about the transcription profile of the bacteria as well as the proteome produced by them is invaluable [95,139,144]. 

Combining empirical and *in-silico* strategies will enable vaccine researchers to benefit from all available information. Genome-based *in-silico* methods in conjunction with proteomics, for example, enable the discovery of cryptic proteins that do not elicit a prominent natural immune response but can nevertheless serve an important role in host-pathogen interaction. Moreover, T-cells may be activated by intra-cellular bacterial proteins, which are not accessible to B-cells and therefore do not elicit an antibody response. The search for such T-cell antigens may be aided by computational approaches. On the other hand, the prediction tools of reverse vaccinology need to be empirically validated. While it may be difficult and of little appeal to test proteins in preclinical models that are anticipated to be useless, immunoproteomics of naturally colonized or infected humans or animals can also serve to test a number of predictions made by the sophisticated bioinformatics tools that are now available to the research community. 

## 5. Future Directions of Research

Omics techniques are developing at a breathtaking pace holding promise for both hypothesis generation and hypothesis testing. The following issues should be addressed in order to apply their full potential to the elucidation of *S. aureus*-host interaction, especially the adaptive immune response, and to vaccine development:

(1) Improvement of important aspects of pre-analytics, such as the rapid enrichment of the pathogen from infected cells and tissues, as well as further increases of the sensitivity of detection methods, should ultimately permit the analysis of pathogen-host interactions directly *ex vivo*.
(2) Interpretation of the “big data” generated by omics techniques relies on sophisticated bioinformatics and depends on the inter-disciplinary dialogue as well as on innovation and technical optimization in the field of computational statistics and bioinformatics.
(3) Prospective clinical trials in well-defined patient cohorts will remain key to finding answers to the burning questions at hand. Such clinical studies should simultaneously consider the pathogen and the immune response, collecting bacterial strains as well as patient sera and immune cells.
(4) Disease specific transcriptome and proteome profiles are required to explain the broad spectrum of diseases caused by the versatile species *S. aureus* and to develop targeted counter-measures.
(5) Advanced omics technologies should be applied to study the adaptive immune response to *S. aureus*. Mapping of antigen, antibody and T-cell repertoires may reveal correlates of protection on which vaccination strategies can then be based.
(6) The multi-omics-approach is very much focused on genes and proteins. Non-protein molecules, however, may be equally important in *S. aureus*-host interaction.

## Figures and Tables

**Table 1:** Overview on omics technologies and their potential applications for deciphering the behavior of *S. aureus* and the host response under infection-relevant conditions.

	Genomics	Transcriptomics	Proteomics	Immunomics	Metabolomics
Omics technologies	2nd generation sequencing methods 3rd generation sequencing methods DNA microarray	Microarray RNAseq	2D-gel-based proteomics in combination with mass spectrometry Gel-free proteomics Protein microarrays	2D-immunoblots Automated 1D immunoblots Suspension arrays Protein arrays Immunocapture mass spectrometry	Mass spectrometry Nuclear magnetic resonance (NMR) spectroscopy
Approaches for deciphering the behavior of *S. aureus*	Sequencing of clinical *S. aureus* isolates Elucidation of the pangenome of the species *S. Aureus* Detection of gene polymorphisms (SNPs, CNVs) Genotyping Population sequencing (e.g., the entire *S. aureus* population in the host organism). T-cell and B-cell epitope prediction	Transcriptomics of clinical *S. aureus* isolates Genome-wide expression profiles of *S. aureus* under infection-relevant conditions *In vivo* transcriptomics Single cell transcriptomics	Proteomics of clinical *S. aureus* isolates Elucidation of the panproteome and subproteomes under infection-relevant conditions *In vivo* proteomics Metaproteomics of the microbiome of the host	Identification of immunogenic, *i.e.*, *in vivo* expressed bacterial antigens	Metabolomics of clinical isolates Metabolomics of bacteria under infection-relevant conditions *In vivo* metabolomics
Approaches for deciphering the host response	Detection of gene polymorphisms (SNPs, CNVs) Genome-wide association studies Analysis of the *S. aureus*-specific T-cell and B-cell repertoires	Genome-wide expression profiles of host cells, e g., immune cells	Proteomics of host cells, e.g., immune cells	Variable and core components of the immunoproteome Monitor antibody profiles upon colonization, infection or vaccination Identification of protective antibody specificities Identification of determinants of antigenicity or the strength of the immune response	Metabolomics of host cells, e.g., immune cells Metabolomics of body fluids
